# Evolutionary analysis of the mTOR pathway provide insights into lifespan extension across mammals

**DOI:** 10.1186/s12864-023-09554-4

**Published:** 2023-08-15

**Authors:** Fei Yang, Xing Liu, Yi Li, Zhenpeng Yu, Xin Huang, Guang Yang, Shixia Xu

**Affiliations:** Jiangsu Key Laboratory for Biodiverity and Biotechnology, College of Life Sciences, Nanjing Normal University, Nanjing, 210023 China

**Keywords:** Mammals, mTOR, Autophagy, Cancer, Longevity

## Abstract

**Background:**

Lifespan extension has independently evolved several times during mammalian evolution, leading to the emergence of a group of long-lived animals. Though mammalian/mechanistic target of rapamycin (mTOR) signaling pathway is shown as a central regulator of lifespan and aging, the underlying influence of mTOR pathway on the evolution of lifespan in mammals is not well understood.

**Results:**

Here, we performed evolution analyses of 72 genes involved in the mTOR network across 48 mammals to explore the underlying mechanism of lifespan extension. We identified a total of 20 genes with significant evolution signals unique to long-lived species, including 12 positively selected genes, four convergent evolution genes, and five longevity associated genes whose evolution rate related to the maximum lifespan (MLS). Of these genes, four positively selected genes, two convergent evolution genes and one longevity-associated gene were involved in the autophagy response and aging-related diseases, while eight genes were known as cancer genes, indicating the long-lived species might have evolved effective regulation mechanisms of autophagy and cancer to extend lifespan.

**Conclusion:**

Our study revealed genes with significant evolutionary signals unique to long-lived species, which provided new insight into the lifespan extension of mammals and might bring new strategies to extend human lifespan.

**Supplementary Information:**

The online version contains supplementary material available at 10.1186/s12864-023-09554-4.

## Background

Lifespan extension has independently evolved several times during mammalian evolution, leading to the emergence of a group of long-lived animals. For example, the bowhead whale (*Balaena mysticetus*) is the longest-lived mammal on the earth with a maximum lifespan (MLS) in excess of 200 years [1], and the African elephant (*Loxodonta africana*) is the longest-lived terrestrial mammal that can live up to 65 years [2]. The naked mole rat (*Heterocephalus glaber*) lives ten times longer than other rodents of similar size [3]. Remarkably, bats can live three times more than other mammals of equal size, and the Brandt’s bat (*Myotis brandtii*) with a body mass of about 7 g but with an MLS of 41 years [4].

Recent studies on these extremely long-lived species indicated that they were equipped with unique mechanisms to extend lifespan. The longest-lived mammal —— the bowhead whale (*B. mysticetus*), was found to extend its lifespan by evolutionary changes associated with DNA repair and insulin signaling pathways, with several DNA repair genes (e.g. *ERCC1*, *ERCC3*, *PCNA*) subject to positive selection and altered expression patterns of a number of insulin signaling (e.g. *GRB14*, *INSIG1*) [1, 5]. Humpback whale (*Megaptera novaeangliae*), MLS of 95 years, possess large segmental duplications in tumor suppressor genes (such as *RPMT2*, *SLC25A6*, *NOX5*) and positive selection genes involved in DNA repair, DNA replication, and cell differentiation [6]. Similarly, the naked mole rat contained the highest copy number of tumor suppressor gene, with an average of 2.39 copies per ortholog among 63 mammals [7]. Importantly, high-molecular-mass hyaluronan was suggested to arrest excessive cell division by early contact inhibition in the naked mole rat. By contrast, the Brandt’s bat was reported to increase the autophagy level with age which could effectively remove aging proteins, organelles, maintain cell homeostasis, and resist age-related diseases [8]. Notably, it would be expected that long-lived animals tend to have increased cancer risk as they have more time to accumulate mutations. However, these species have been reported to have lower incidence of cancer than short-lived species [9]. For example, naked mole rats have the extraordinary cancer resistance, as a long-term observation of naked mole rats (*n* = 800) did not detect a single incidence of cancer [10]. Thus, these extremely long-lived mammals might have evolved specific mechanisms of cancer resistance to extend lifespan, however, the underlying common mechanisms are still poorly explored. The mechanistic/mammalian target of rapamycin (mTOR) plays a key role in regulating lifespan with reduced mTOR expression and activity extending lifespan in model organisms [11]. The mTOR protein kinase nucleates a major eukaryotic signaling network that senses and integrates environmental and intracellular nutrient and growth factor signals to coordinate many fundamental cell processes, from protein synthesis to autophagy [12]. Extensive research have revealed that dysregulated mTOR network is implicated in the progression of cancer and diabetes, as well as the aging process [13]. Thus, the mTOR network can be an ideal target for studying its influence on the evolution of lifespan in mammals.

mTOR functions in two distinct complexes, known as mTOR Complex 1 (mTORC1) and 2 (mTORC2) [14]. mTORC1, which senses multiple upstream signals such as amino acids and hypoxia, and is involved in protein synthesis, lipid metabolism [15], regulates autophagy through interacting with a series of complexes, including V-ATPase, Rag-Ragulator, etc. Autophagy is a process in which cells process excess or defective organelles and other macromolecular substances through lysosomes, to avoid the harmful effects of proteins and organelles accumulated with age and ensure the metabolic homeostasis of cells [16, 17]. Genetic evidence indicates that autophagy has crucial roles in lifespan regulation, and the basal level of autophagy activity is increased in many long-lived species [18]. Recent researches have revealed that genetic change of ATPase or Rag-Ragulator resulted in neurodegenerative diseases, such as Alzheimer’s disease and Parkinson's disease [19, 20], which were closely related to the dysfunction of autophagy. Moreover, other key upstream regulators including TSC complex and *AKT* were well-known cancer related genes [13]. By contrast, mTORC2 shares two key subunits (*MLST8* and *SEC13*) with mTORC1, and regulates cell survival, cytoskeletal organization. Importantly, hyperactivated mTORC2 and its substrates were also commonly involved in tumorigenesis [21, 22].

In this study, we conducted an evolutionary analysis of 72 genes involved in the mTOR pathway network across 48 mammalian lineages to explore the potential mechanism of extending lifespan. Here, we mainly aimed to investigate 1) whether mTOR network underwent different evolutionary patterns between long-lived mammals and the control group, 2) whether gene evolution rate related to longevity associated traits, such as MLS, BM, and longevity quotient (LQ) — MLS after BM correction, 3) whether different long-lived species shared convergent amino acid mutations in this network. Based on these results, we hope to provide new insights into mechanism of the mTOR pathway on lifespan evolution across mammals.

## Results

### Identification of long-lived species

**Fig. 1:**
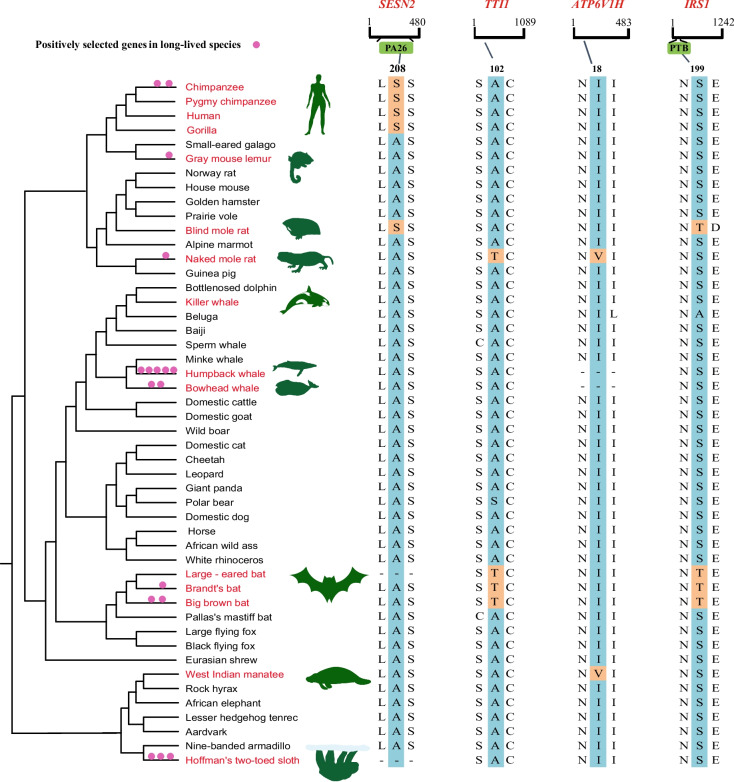
Evidence of positive selection across phylogeny of mammals identified by branch-site model and BUSTED. Long-lived species identified by LQ > 1.57 are highlight in red. Phylogenetic tree of 48 mammals is taken from TimeTree database. Illustrations of long-lived species are taken from phylopic.org. Positively selected genes identified by branch-site model and BUSTED are indicated by pink circles. Convergent amino acid changes occurred in long-lived are shown on the right of the figure (the convergence sites in long-lived species are marked in orange and the remaining are in blue)

We calculated LQ value for each species using MLS and BM obtained from AnAge database based on the allometric equation: LQ=MLS/(6.32*BM^0.139). In reference to the study of Yu et al. [23], mean LQ ± 1SD (1 ± 0.57) was used as the standard to classify long-lived species and control group. In our dataset, 15 species with LQ > 1.57 were classified as long-lived, including the human (*Homo sapiens*), gorilla (*Gorilla gorilla*), chimpanzee (*Pan troglodytes*), pygmy chimpanzee (*P. paniscus*), gray mouse lemur (*Microcebus murinus*), naked mole-rat (*Heterocephalus glaber*), west Indian manatee (*Trichechus manatus*), brandt's bat (*Myotis brandtii*), large-eared bat (*Myotis myotis*), big brown bat (*Eptesicus fuscus*), blind mole rat (*Nannospalax galili*), bowhead whale (*Balaena mysticetus*), killer whale (*Orcinus orca*), humpback whale (*Megaptera novaeangliae*), hoffmann's two-toed sloth (*Choloepus hoffmanni*) (Fig. 1, Table S1). And the remaining species with LQ < 1.57 were classified as the control group.

### Positive selection test of the mTOR network unique to long-lived mammals

**Fig. 2:**
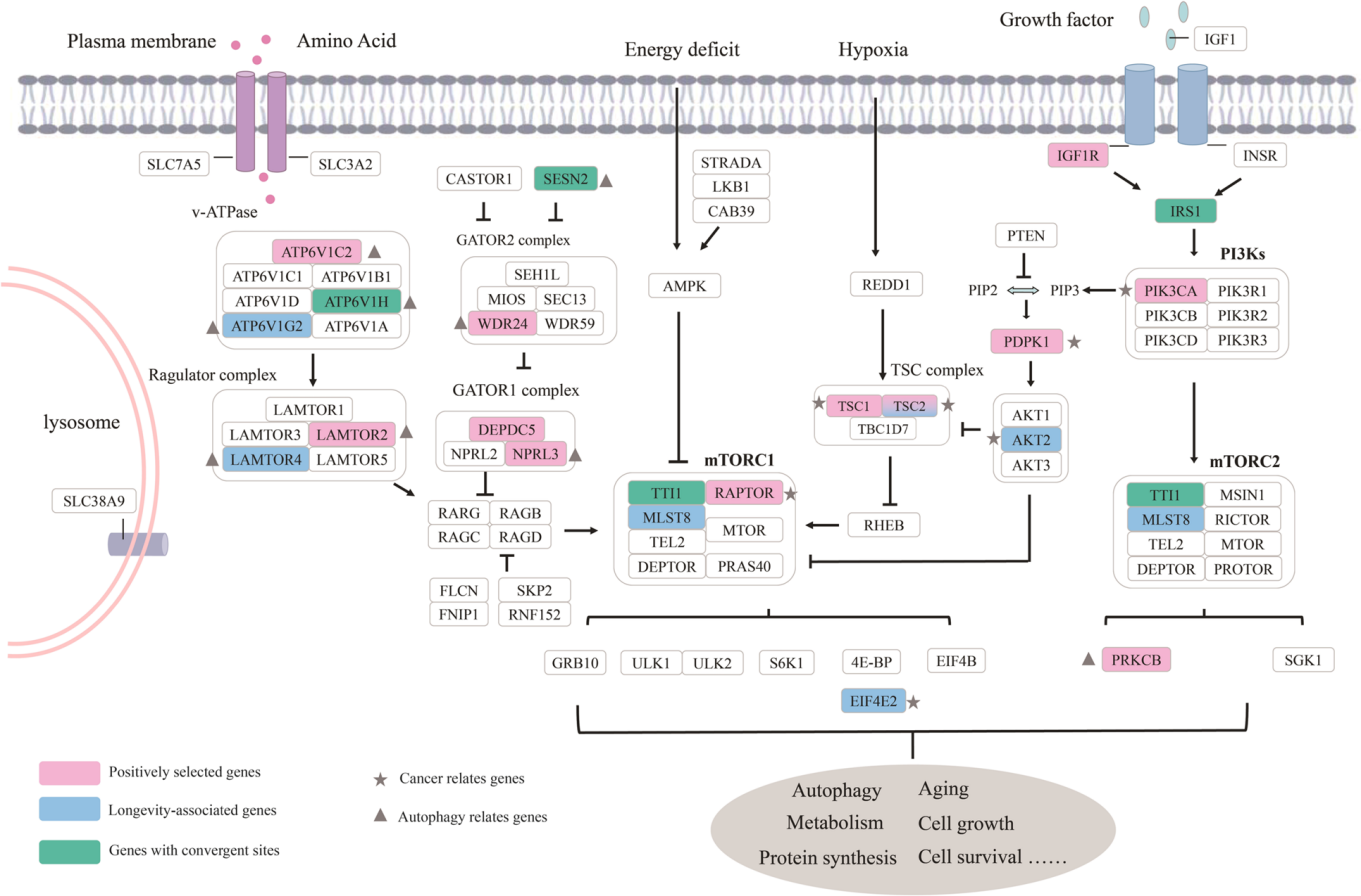
Genes with significant evolution signals identified in long-lived species. Positively selected genes, convergent evolution genes and longevity-associated genes are marked in pink, green and blue, respectively. Genes related to cancer and autophagy are shown by gray five-pointed star and gray triangle, respectively

We first detected positive selection in long-lived species using a stringent branch-site model, as implemented in CODEML from the PAML 4.9e. The result revealed that 23.61% of genes (17/72) showed signals of positive selection in at least one long-lived lineage after FDR correction for multiple tests (Table S2). In addition, 70 sites were examined to be under positive selection using the BEB approach with posterior probabilities > 0.80 (Table S2). Positive selection was further validated using BUSTED in Hyphy that may boost power of positive selection detection if only one or a few amino acid sites are under selection in the entire gene. The result showed that 16 genes were subject to positive selection (Table S3). A total of 12 genes under positive selection were identified by both branch-site tests (branch-site model and BUSTED) in long-lived species, including *TSC2*, *TSC1*, *RAPTOR*, *PIK3CA*, *PDPK1*, *ATP6V1C2*, *WDR24*, *DEPDC5*, *NPRL3*, *LAMTOR2*, *IGF1R* and *PRKCB*. We further assessed whether these 12 positively selected genes were also shared in the control group. We performed a pair of site model, i.e. M8 vs M8a, in the dataset that was restricted to the control group. Likelihood ratio tested (LRTs) showed that M8 including sites with ω > 1 fitted significantly better than the neutral model (M8a) at five genes (*SLC3A2*, *FNIP1*, *SLC38A9*, *ATP6V1D* and *STRADB*, Table S4), with 17 codons identified to be under positive selection based on BEB analysis with posterior probabilities ≥ 0.80. The five genes did not overlap with the 12 positively selected genes identified by both branch-site tests. Thus, 12 genes (*TSC2*, *TSC1*, *RAPTOR*, *PIK3CA*, *PDPK1*, *ATP6V1C2*, *WDR24*, *DEPDC5*, *NPRL3*, *LAMTOR2*, *IGF1R* and *PRKCB*, Fig. 2) were considered as candidates for positive selection unique to the long-lived species.

We also conducted a positive selection test on random-set genes unique to long-lived mammals to determine if a similar pattern exists in genes other than the mTOR pathway, and the analyses revealed that no signals of positive selection were detected in long-lived lineages (Table S5.1, Table S5.2 and Table S5.3).

### Association between gene evolution and longevity-associated traits

**Fig. 3:**
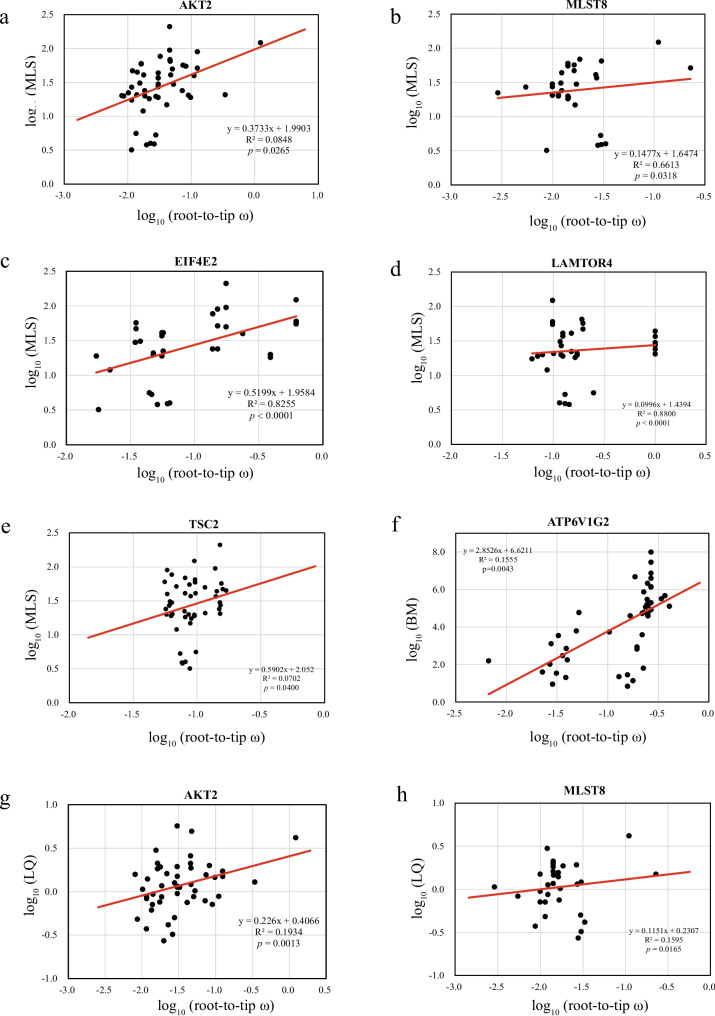
Regression analyses between root-to-tip ω and morphological variables (MLS, LQ and BM)

PGLS analysis was used to address the link between evolutionary rates and longevity-associated traits (MLS, BM and LQ). In mTOR pathway, result revealed that evolutionary rates (root-to-tip ω) (Table S6) were significantly related to MLS at the five genes, including *MLST8* (R^2^ = 0.6613, *p* = 0.0318), *LAMTOR4* (R^2^ = 0.8800, *p* < 0.0001), *EIF4E2* (R^2^ = 0.8255, *p* < 0.0001), *AKT2* (R^2^ = 0.0848, *p* = 0.0265), and *TSC2* (R^2^ = 0.0702, *p* = 0.0400, Fig. 3, Table S7). Of them, molecular evolution of both *AKT2* (R^2^ = 0.1934, *p* = 0.0013) and *MLST8* (R^2^ = 0.1595, *p* = 0.0165) were also significantly correlated with LQ (Fig. 3, Table S7). A significant association between evolutionary rates and BM were examined at *ATP6V1G2* (R^2^ = 0.1555, *p* = 0.0043) (Fig. 3, Table S7). Notably, the evolutionary rates of both *AKT2* and *MLST8* showed an association with both MLS and LQ. In the random-gene dataset, we found that the evolutionary rate of one gene was significantly correlated with BM (Table S8.1).

### Convergent amino acid changes in long-lived group

To search for convergent evolution in long-lived mammals, we scanned all homologous genes using the FasParser2 software. Five substitutions were identified in long-lived species, including *SESN2* (A208S) in four primates (Human, Gorilla, Chimpanzee, Pygmy chimpanzee) and the blind mole rat, *MIOS* (A378T) in three bats (Brandt's bat, Large-eared bat and Big brown bat) and four primates, *ATP6V1H* (I18V) in the naked mole rat and manatee, *IRS1* (S199T) shared by the blind mole rat and three bats, while *TTI1* (A102T) shared by the naked mole rat and three bats (Fig. 1). Importantly, four amino acid mutations of *SESN2*, *IRS1*, *TTI1*and *ATP6V1H* are still unique to the long-lived groups when expanding the dataset to 100 mammals. In addition, the four amino acid mutations were also shared by the newly identified long-lived species according to our criteria, such as site 102 of *TTI1* shared by *Myotis lucifugus* (LQ = 3.91) and site 208 of *SESN2* shared by *Cebus capucinus* (LQ = 2.86) (Figure S1, Table S9)*.* Moreover, we find that two convergent sites of *SESN2* and *IRS1* are in the key protein domains identified by Pfam database [24] (Table S10). However, the mutation of *MIOS* (A378T) was also examined in the control group, such as *Phascolarctos cinereus* and *Notamacropus eugenii* (Table S9). Taken together, the four long-lived-specific convergent amino acid changes were identified in our study, including *SESN2* (A208S), *ATP6V1H* (I18V), *IRS1* (S199T) and *TTI1* (A102T).

In our study, we found a total of 20 genes show significant evolutionary signals in long-lived species, and eight out of the 20 genes showing significant evolutionary signals were listed as cancer genes in the OncoKB database [25], including *TSC1*, *TSC2*, *PIK3CA*, *RAPTOR*, *IGF1R*, *PDPK1*, *PRKCB* and *AKT2*.

## Discussion

Maximum lifespans differ up to 100-fold between extant mammalian species, ranging from 211 years in the bowhead whale to 2.1 years in the forest shrew (*Myosorex varius*) [26]. Recent studies have shown that extremely long-lived species, such as African elephants, bowhead whales, brandt's bats and naked mole rats, have evolved their unique mechanisms of lifespan extension. However, the shared mechanisms underlying mammalian longevity are complex and still not fully understood. In this study, we performed molecular evolutionary analyses of 72 genes involved in mTOR network across 48 mammals and identified some potential mechanisms underlying extended lifespan. Our results revealed 12 positively selected genes (*TSC2*, *TSC1*, *RAPTOR*, *PIK3CA*, *PDPK1*, *ATP6V1C2*, *WDR24*, *DEPDC5*, *NPRL3*, *LAMTOR2*, *IGF1R* and *PRKCB*) and four convergent amino acid changes involved in four genes (*SESN2*, *ATP6V1H*, *IRS1* and *TTI1*) were unique to long-lived species. Evolutionary rates of five genes (*MLST8*, *LAMTOR4*, *EIF4E2*, *AKT2* and *TSC2*) were significantly related to the longevity-associated traits. Combined, a total of 20 genes (with one overlapped gene, *TSC2*) were identified to have significant evolution signs in the long-lived mammals, which may help explain the role of mTOR pathway in the evolution of mammalian lifespan extension. Moreover, the analyses conducted on a control dataset of other genes showed that the genes in the mTOR pathway exhibit positive selection signals in long-lived lineages more significantly (Fisher's exact test *p* = 2.016e-05), providing further evidence to support this conclusion.

### Enhanced autophagy to resist aging-related diseases in long-lived mammals

One hallmark of aging is the accumulation of various damaged organelles, misfolded protein or DNA mutations, as well as the incidence of chronic diseases such as neurodegeneration, which rises with age [16]. Autophagy is a conserved physiological process that relies on lysosomes to degrade intracellular macromolecules (such as excess or defective proteins and organelles), which is essential for the organism to maintain its homeostasis [17]. It was shown that enhanced autophagy was associated with extended lifespan in model organisms (such as worm and mouse) [18], and increasing evidences indicated that several genes (including *LAMTOR4*, *LAMTOR2* and *NPRL3*) involved in mTOR network regulated autophagy and neurodegenerative diseases [27, 28].

In this study, we found that four positively selected genes (*PRKCB*, *WDR24*, *NPRL3* and *LAMTOR2*) and one longevity-associated gene (*LAMTOR4*) in long-lived species were involved in the autophagy response and aging-related diseases. LAMTOR (also known as Ragulator) is a scaffold protein complex that regulates mTOR signaling responding to amino acid signals, thus influencing cell growth and proliferation. *LAMTOR2*, a subunit of LAMTOR, is involved in autophagy and functions as a selective autophagy regulator by mediating autophagosome–lysosome fusion [29]. Similarly, *NPRL2* and *NPRL3* were also involved in regulation of autophagy as deletions of both homologous genes resulted in significant defects in nitrogen-starvation-induced autophagy in yeast [30]. Researches from fruit flies and zebrafish have demonstrated that *WDR24* plays a role in regulating autophagy initiation [31]. For instance, *WDR24* knockdown results in developmental defects, at least in part, due to dysregulated autophagy, which could be partially restored by *WDR24* overexpression [32]. By contrast, overexpression of *PRKCB* leads to an inhibition of autophagy [33]. Moreover, of five longevity-associated genes identified in our study, *LAMTOR4* was revealed associated with aging diseases. *LAMTOR4* mutants reduced the number of phagocytic cells — microglia, leading to weakened phagocytosis and reduced the clearance of Aβ protein in Alzheimer's disease [34]. There is increasing evidence that autophagy is an effective neuroprotective mechanism and malfunctioning autophagy frequently leads to neurodegenerative diseases, such as Parkinson's disease or Alzheimer's disease [35]. Collectively, enhanced autophagy to resist aging-related diseases may be an important mechanism of life extension in long-lived mammals.

### Lifespan extension through modulating cancer and aging genes

Previous studies have shown that several extremely long-lived species have evolved unique cancer resistance mechanisms to extend their lifespan. For instance, the naked mole rat's immunity to cancer was attributed to the cell contact inhibition caused by high-molecular-mass hyaluronan, while it was believed that the bland mole rat's tumor transformation was prevented by interferon-mediated 'concerted cell death'. [36]. Genes included in OncoKB can influence mammalian lifespan through various mechanisms. For example, *TSC2* (tuberous sclerosis complex 2) was a tumor suppression gene that was reported that specific mutations of this gene led to the development of renal and extra-renal tumors in mice [37]. Moreover, *TSC1* and *TSC2* form tumor suppressor complex to control cell growth and prevent disease [38]. In our study, both *TSC1* and *TSC2* genes were subject to positive selection special for long-lived species, and *TSC2* evolutionary rate was significantly related to MLS. Another long-lived mammals-specific positive selection gene—*RAPTOR* (Regulatory associated protein of mTOR complex 1) was responsible for the induction of mTORC1 during tumorigenesis and progression, such as *RAPTOR* overexpression in colorectal cancer tissues and cell lines [39]. Notably, of the eight cancer-related genes, six (*TSC1*, *TSC2*, *PIK3CA*, *IGF1R*, *PDPK1* and *AKT2*) are known longevity related genes according to GenAge database in human/model organisms [26], and genetic manipulation of these genes can directly lead to shortening or extending lifespan of model organisms. For instance, knockout of *daf-2* (homologous to *IGF1R* in mammals) in nematodes (*Caenorhabditis elegans*), leads to doubled lifespan extension [40]. Overexpression of *Tsc1* and *Tsc2* in *Drosophila *extended mean lifespan by 14% and 12%, respectively [41]. As observed above, a number of genes related to cancer and aging were examined to have significant evolution signals in long-lived species, suggesting that mammals might increase lifespan by regulating cancer and aging related genes. However, nearly half of genes with evolution signals in our study are oncogenes. This pattern could be explained by previous findings that these genes play a critical role in the tradeoff between living longer and disease susceptibility later in life [42]. And this also suggests that the evolution of the mTOR signaling pathway may play a crucial role in longevity of species.

### Convergent autophagy in long-lived species

Convergent autophagy in long-lived species could partially modulate increased longevity. Our results exhibited that there were four convergent amino acid mutations at four genes (*ATP6V1H*, *TTI1*, *IRS1*, *SESN2*) in deeply distant long-lived species: the naked mole rat, blind mole rat, manatee, bats (Brandt's bat, Large-eared bat and Big brown bat) and primates (Human, Gorilla, Chimpanzee, Pygmy chimpanzee). Of them, three convergent amino acid sites of three genes are located in key function domains (*SESN2*, *ATP6V1H* and *IRS1*). *ATP6V1H* was proven to participate in the formation of phagosomes and a recent comparative transcriptomes analysis showed that *ATP6V1H* was highly expressed in long-lived bats [8]. *SESN2*, known as stress-inducible protein, may function in the regulation of cell growth, survival and autophagy [43], overexpression of *SESN2* can promote autophagy in neuroblastoma cells [44]. *TTI1* is a highly conserved regulator of DNA damage response, which can maintain the stability of genome and therefore extend lifespan [45]. Two convergent genes in long-lived species in our study are strongly associated with autophagy, suggesting convergent autophagy might be another mechanism to extend lifespan in long-lived mammals. Of course, more experiments are needed to explore if the convergent site of autophagy gene contributes to longevity in the future.

## Conclusions

Understanding the mechanisms responsible for the dramatic differences in lifespan between species will have a transformative effect on developing interventions to improve human health and longevity. In our study, molecular evolution analyses of genes involved in the mTOR network were examined across mammals to reveal the mechanisms of lifespan extension. Autophagy related genes were identified to be under positive selection (*PRKCB*, *WDR24*, *NPRL3* and *LAMTOR2*) or convergent (*ATP6V1H* and *SESN2*) in long-lived species, or associated with MLS (*LAMTOR4*), suggesting that enhanced autophagy might be a potential mechanism for mammals to extend lifespan. Moreover, eight genes with evolutionary signals identified in long-lived species were cancer related genes, six of them were also associated with aging, suggesting that regulation of cancer and aging may be another important mechanism for extending lifespan. In conclusion, we identified 20 genes with significant evolutionary signals unique to long-lived species, which provided new insight into the lifespan extension of mammals and might bring new strategies to extend human lifespan.

## Methods

### Phenotype data collection and identification of long-lived species

In this study, we selected 48 mammalian species (representing 14 orders) including extremely long-lived species and their relatives. Then, we collect longevity associated phenotype data (i.e. MLS and BM) of 48 species from the AnAge database [26] (https://genomics.senescence.info/species/). To rule out the effect of body mass on MLS, we used longevity quotient (LQ) — the ratio of the observed MLS to the predicted MLS — to define whether a species is long-lived. The LQ values were calculated according to the allometric equation: LQ=MLS/(6.32*BM^0.139) [46]. Then we classified long-lived species referring to a recent study of Yu et al. [23] where species with LQ greater than 1 SD (standard deviation) were identified as long-lived species, while others were classified as control group (Table S1).

### Sequence retrieval and alignments

A total of 72 well-annotated key genes of human mTOR network were drawn from the Kyoto Encyclopedia of Genes and Genomes (KEGG) database [47] (https://www.kegg.jp/pathway/hsa04150) (Table S11). Then protein-coding sequences of the other 48 mammals were downloaded from the OrthoMAM v10 database (https://neworthomam.mbb.univ-montp2.fr/) [48] and the Bowhead Whale Genome Resource (http://www.bowhead-whale.org/). When gene sequences are not available in public database, we downloaded mammalian genomes from the NCBI database (https://www.ncbi.nlm.nih.gov/genome/) (Table S1) and performed local BLAST (blastn -evalue 1e-6) [49] to search sequences using human orthologs as queries. Moreover, in order to determine if the inferred selective patterns are unique to the mTOR pathway rather than a genome-wide phenomenon, we expanded positive selection analyses of 60 random-set genes in the long-lived mammals (Table S12). Coding sequences of each gene were aligned using PRANK (-codon -F, prank v.170427) [50] at the codon level, and then removed the low-homologous region and poorly aligned regions with gaps using Gblocks (-t = c -b1 = 25 -b5 = h, version 0.91b) [51]. High quality multiple sequence alignments of 72 genes involved in mTOR network and random-gene dataset were used for the subsequent analysis.

### Molecular evolution analyses

Selective pressure acting on protein-coding genes is often detected by estimating the ratio (ω) of nonsynonymous (*d*_N_) to synonymous (*d*_S_) substitutions. Values of ω > 1, = 1 and < 1 indicate positive selection, neutral evolution and purifying selection, respectively. We used two independent tests for positive selection: the branch-site model of the PAML 4.9e suite [52, 53] and the branch-site unrestricted test for episodic diversification (BUSTED) implemented in HyPhy (version 3.0) [54, 55]. Both methods assume that all branches in the tree can be partitioned a priori into the foreground branches (interested) and the background branches. The PAML branch-site test compares a model (MA) that allows positive selection on foreground lineages with a null model (MA1) that does not allow such positive selection. Positively selected sites were tested using the Bayes Empirical Bayes (BEB) analysis with posterior probabilities > 0.80. By contrast, BUSTED includes three ω classes, but differs from PAML (ω_1_ ≤ ω_2_ ≤ 1 ≤ ω_3_) with ω_3_ > 1 for all foreground branches in the unconstrained model (alternative model), and ω_3_ = 1 in all foreground branches in the null model [55]. Putative positive selection was tested by both branch-site tests focusing on each foreground branch, i.e. the long-lived lineage. Both methods apply likelihood ratio tests (LRT) to assess the fitness of the alternative model. A false discovery rate (FDR) correction for multiple tests was conducted in both methods. A well-accepted phylogenetic tree of 48 species from the TimeTree database (http://www.timetree.org/) was used as the input tree for the analyses.

To exclude the false positive selection events in the foreground branches with ω > 1 also in the background branches, we removed foreground branches and run a pair of site model, i.e. M8a (neutral; beta distribution: 0 < ω_0_ < 1 and ω1 = 1) vs M8 (positive selection; beta distribution: 0 < ω_0_ < 1 and ω1 > 1) [56] to identify possible positive selection sites in each gene. Finally, we filter out genes with evidence for positive selection identified by the site model from positively selected genes detected by both branch-site tests in the foreground branches. Genes that show evidence of positive selection in long-lived species from both PAML branch-site model and HyPhy BUSTED and show no signs of positive selection from site-model in the non-long-lived species are considered as candidates for positive selection unique to long-lived species.

### Association analysis between gene evolution and phenotypes

We perform phylogenetic generalized least squares (PGLS) regression to evaluate the possible relationship between evolutionary rates (ω) of mTOR pathway genes (Table S6), random-set genes (Table S8.2) and longevity associated traits, including MLS, BM and LQ. The λ value estimated by the maximum likelihood method is used to quantitatively evaluate the intensity of phylogenetic signals [57]. λ ranges from 0 to 1, with 0 indicating the absence of phylogenetic signals, and λ close to 1 indicating the presence of a strong phylogenetic signal. The root-to-tip ω of each gene was estimated in free-ratio model implemented in the CODEML program in PAML 4.9e [52]. All longevity variables (MLS, BM, LQ) and root-to-tip ω were transformed to log10. All statistical analyses were performed in the “Caper” package in R 3.4.2 [58].

### Convergent amino acid site shared between long-lived mammals

To investigate the potential effect of convergent amino acid change of genes involved in mTOR network in long-lived mammals, we scanned for the same amino acid changes between all pairs of long-lived groups and differ from the other lineages using FasParser2 [59]. The shared amino acid sites in the two long-lived groups (primates, bats, naked mole rat, blind mole rat, west Indian manatee and cetaceans) that are different from the rest of the species are considered as convergent sites. To further verify the reality of these convergent signatures, we expand our dataset to 100 mammals. Then we used the human amino acid sequences to correct the positions of these convergent amino acid mutations, and Pfam database [24] was used to determine whether these sites were located in the important functional domain of the protein.

### Supplementary Information

**Additional file 1:** **Figure S1.** Other mammals share same amino acid sites with long-lived species in this study.

**Additional file 2**: **Table S1.** Longevity-associated traits of 48 mammals used in the present study. **Table S2.** Positively selected genes identified based on the branch-site model of PAML in mTOR pathway. **Table S3.** Genes inferred to be under positive selection using BUSTED models in mTOR pathway. **Table S4.** Selective pressure analyses of mTOR pathway genes by site model. **Table S5.** Results of selective pressure analyses of random-set genes. **Table S6.** Root-to-tip ω of mTOR pathway genes used in this study. **Table S7.** Summary of mTOR pathway genes with a root-to-tip *dN*/*dS* significantly correlated with maximum lifespan (MLS), longevity quotient (LQ), body mass (BM). **Table S8.** Random-set genes correlated with longevity associated traits and root-to-tip ω values of random-set genes. **Table S9.** Longevity-associated traits of other species with the same convergent sites as the long-lived species in this study. **Table S10.** Protein domains of convergent amino sites. **Table S11.** List of mTOR pathway genes used in the present study. **Table S12.** Random-set genes list.

## Data Availability

The data generated and analyzed during this study are included in this article and its additional files, including 12 tables and 4 figures. Gene ids in OrthoMaM database are available in Table S11, genome versions of NCBI database in this study are available in Table S1. Genes of mTOR pathway are taken from: https://www.kegg.jp/pathway/hsa04150 Other important links for additional data: https://neworthomam.mbb.univ-montp2.fr/ http://www.bowhead-whale.org/ https://www.ncbi.nlm.nih.gov/genome/ https://genomics.senescence.info/species/

## Abbreviations

MLS: Maximum lifespan
BM: Body mass
LQ: Longevity quotient
PAML: Phylogenetic analysis by maximum likelihood
BLAST: Basic local alignment search tool
PGLS: Phylogenetic generalized least squares
LRT: Likelihood ratio test
FDR: False discovery rate
SD: Standard deviation
BUSTED: Branch-Site Unrestricted Statistical Test for Episodic Diversification
