# Supplementary material for: Evolutionary analysis of the mTOR pathway provide insights into lifespan extension across mammals
Source: BMC Genomics. 2023 Aug 15;24:456. doi: 10.1186/s12864-023-09554-4 (PMC10426088; doi:10.1186/s12864-023-09554-4)
Supplement: Supplementary file 1 — Additional file 1: Figure S1. Other mammals share same amino acid sites with long-lived species in this study. [file 12864_2023_9554_MOESM1_ESM.pdf]

SESN2			
Nannospalax galili	L	S	S
Homo sapiens	L	S	S
Gorilla gorilla	L	S	S
Pan paniscus	L	S	S
Pan troglodytes	L	S	S
Saimiri boliviensis	L	S	S
Nomascus leucogenys	L	S	S
Callithrix jacchus	L	S	S
Cebus capucinus	L	S	S
Macaca nemestrina	L	S	S
Macaca fascicularis	L	S	S
Macaca mulatta	L	S	S
Capra hircus	L	A	S
Loxodonta africana	L	A	S
Procavia capensis	L	A	S
Lipotes vexillifer	L	A	S
Orycteropus afer	L	A	S
Cavia aperea	L	A	S
Chinchilla lanigera	L	A	S
Hipposideros armiger	L	A	S
Mus caroli	L	A	S
Myotis davidii	L	A	S
Monodelphis domestica	L	A	S
Notamacropus eugenii	L	A	S
Phascolarctos cinereus	L	A	S
Ailuropoda melanoleuca	L	A	S
Ursus maritimus	L	A	S
Enhydra lutris	L	A	S
Canis familiaris	L	A	S
Odobenus rosmarus	L	A	S
Neomonachus schauinslandi	L	A	S
Leptonychotes weddellii	L	A	S
Mustela putorius	L	A	S
Galeopterus variegatus	L	A	S
Otolemur garnettii	L	A	S
Ictidomys tridecemlineatus	L	A	S
Microcebus murinus	L	A	S
Propithecus coquereli	L	A	S
Carlito syrichta	L	A	S
Condylura cristata	L	A	S
Ceratotherium simum	L	A	S
Equus caballus	L	A	S
Equus przewalskii	L	A	S
Equus asinus	L	A	S
Acinonyx jubatus	L	A	S
Panthera pardus	L	A	S
Panthera tigris	L	A	S
Felis catus	L	A	S
Bos taurus	L	A	S
Bos indicus	L	A	S
Bos mutus	L	A	S
Bison bison	L	A	S
Bubalus bubalis	L	A	S
Odocoileus virginianus	L	A	S
Ovis aries	L	A	S
Pantholops hodgsonii	L	A	S
Balaenoptera acutorostrata	L	A	S
Physeter catodon	L	A	S
Orcinus orca	L	A	S
Delphinapterus leucas	L	A	S
Tursiops truncatus	L	A	S
Sus scrofa	L	A	S
Camelus bactrianus	L	A	S
Camelus dromedarius	L	A	S
Camelus ferus	L	A	S
Jaculus jaculus	L	A	S
Dipodomys ordii	L	A	S
Castor canadensis	L	A	S
Cavia porcellus	L	A	S
Octodon degus	L	A	S
Heterocephalus glaber	L	A	S
Fukomys damarensis	L	A	S
Rousettus aegyptiacus	L	A	S
Pteropus alecto	L	A	S
Pteropus vampyrus	L	A	S
Miniopterus natalensis	L	A	S
Rhinolophus sinicus	L	A	S
Dasypus novemcinctus	L	A	S
Chrysochloris asiatica	L	A	S
Manis javanica	L	A	S
Mesocricetus auratus	L	A	S
Rattus norvegicus	L	A	S
Cricetulus griseus	L	A	S
Meriones unguiculatus	L	A	S
Microtus ochrogaster	L	A	S
Peromyscus maniculatus	L	A	S
Mus musculus	L	A	S
Mus spretus	L	A	S
Mus pahari	L	A	S
Elephantulus edwardii	L	A	S
Trichechus manatus	L	A	S
Eptesicus fuscus	L	A	S
Myotis lucifugus	L	A	S
Myotis brandtii	L	A	S
Ochotona princeps	L	A	S
Oryctolagus cuniculus	L	A	S
Erinaceus europaeus	L	A	S
Sorex araneus	L	A	S
Sarcophilus harrisii	L	A	S
Ornithorhynchus anatinus	L	A	S

MIOS			
Myotis myotis	C	T	T
Eptesicus fuscus	C	T	T
Myotis brandtii	C	T	T
Homo sapiens	C	T	T
Pan paniscus	C	T	T
Pan troglodytes	C	T	T
Gorilla gorilla	C	T	T
Myotis lucifugus	C	T	T
Mandrillus leucophaeus	C	T	T
Cebus capucinus	C	T	T
Macaca nemestrina	C	T	T
Colobus angolensis	C	T	T
Macaca fascicularis	C	T	T
Callithrix jacchus	C	T	T
Saimiri boliviensis	C	T	T
Nomascus leucogenys	C	T	T
Macaca mulatta	C	T	T
Oryctolagus cuniculus	C	T	T
Erinaceus europaeus	C	T	T
Sarcophilus harrisii	C	T	T
Phascolarctos cinereus	C	T	T
Notamacropus eugenii	C	T	T
Ailuropoda melanoleuca	C	A	A
Canis familiaris	C	A	A
Ictidomys tridecemlineatus	C	A	A
Odobenus rosmarus	C	A	A
Ursus maritimus	C	A	A
Marmota marmota	C	A	A
Panthera pardus	C	A	A
Panthera tigris	C	A	A
Hipposideros armiger	C	A	A
Condylura cristata	C	A	A
Cavia porcellus	C	A	A
Rhinolophus sinicus	C	A	A
Cricetulus griseus	C	A	A
Microtus ochrogaster	C	A	A
Peromyscus maniculatus	C	A	A
Rattus norvegicus	C	A	A
Enhydra lutris kenyoni	C	A	A
Neomonachus schauinslandi	C	A	A
Leptonychotes weddellii	C	A	A
Acinonyx jubatus	C	A	A
Miniopterus natalensis	C	A	A
Dipodomys ordii	C	A	A
Microcebus murinus	C	A	A
Orcinus orca	C	A	A
Balaenoptera acutorostrata	C	A	A
Delphinapterus leucas	C	A	A
Lipotes vexillifer	C	A	A
Tursiops truncatus	C	A	A
Ceratotherium simum	C	A	A
Galeopterus variegatus	C	A	A
Trichechus manatus latirostris	C	A	A
Camelus dromedarius	C	A	A
Camelus bactrianus	C	A	A
Camelus ferus	C	A	A
Propithecus coquereli	C	A	A
Loxodonta africana	C	A	A
Otolemur garnettii	C	A	A
Mus caroli	C	A	A
Mus musculus	C	A	A
Mus spretus	C	A	A
Mus pahari	C	A	A
Mustela putorius	C	A	A
Physeter catodon	C	A	A
Jaculus jaculus	C	A	A
Sus scrofa	C	A	A
Manis javanica	C	A	A
Bos taurus	C	A	A
Bison bison bison	C	A	A
Bos indicus	C	A	A
Bubalus bubalis	C	A	A
Odocoileus virginianus	C	A	A
Bos mutus	C	A	A
Ovis aries	C	A	A
Pantholops hodgsonii	C	A	A
Capra hircus	C	A	A
Tupaia belangeri	C	A	A
Tupaia chinensis	C	A	A
Felis catus	C	A	A
Meriones unguiculatus	C	A	A
Orycteropus afer	C	A	A
Elephantulus edwardii	C	A	A
Pteropus alecto	C	A	A
Rousettus aegyptiacus	C	A	A
Equus przewalskii	C	A	A
Equus asinus	C	A	A
Equus caballus	C	A	A
Cavia aperea	C	A	A
Sorex araneus	C	A	A
Castor canadensis	C	A	A
Fukomys damarensis	C	A	A
Ochotona princeps	C	A	A
Procavia capensis	C	A	A
Dasypus novemcinctus	C	A	A
Mesocricetus auratus	C	A	A
Choloepus hoffmanni	C	A	A
Chinchilla lanigera	C	A	A
Chrysochloris asiatica	C	A	A
Echinops telfairi	C	A	A

ATP6V1H			
Trichechus manatus	N	V	I
Heterocephalus glaber	N	V	I
Panthera tigris	N	I	I
Microcebus murinus	N	I	I
Otolemur garnettii	N	I	I
Castor canadensis	N	I	I
Meriones unguiculatus	N	I	I
Ailuropoda melanoleuca	N	I	I
Rhinolophus sinicus	N	I	I
Canis familiaris	N	I	I
Orycteropus afer	N	I	I
Equus caballus	N	I	I
Mustela putorius	N	I	I
Ursus maritimus	N	I	I
Enhydra lutris	N	I	I
Equus asinus	N	I	I
Rousettus aegyptiacus	N	I	I
Miniopterus natalensis	N	I	I
Neomonachus schauinslandi	N	I	I
Odobenus rosmarus	N	I	I
Dasypus novemcinctus	N	I	I
Loxodonta africana	N	I	I
Sus scrofa	N	I	I
Balaenoptera acutorostrata	N	I	I
Pteropus alecto	N	I	I
Eptesicus fuscus	N	I	I
Physeter catodon	N	I	I
Myotis davidii	N	I	I
Chrysochloris asiatica	N	I	I
Felis catus	N	I	I
Panthera pardus	N	I	I
Tursiops truncatus	N	I	I
Orcinus orca	N	I	I
Lipotes vexillifer	N	I	I
Delphinapterus leucas	N	I	I
Myotis lucifugus	N	I	I
Myotis brandtii	N	I	I
Condylura cristata	N	I	I
Elephantulus edwardii	N	I	I
Ceratotherium simum	N	I	I
Bos taurus	N	I	I
Bison bison bison	N	I	I
Bos indicus	N	I	I
Bubalus bubalis	N	I	I
Pantholops hodgsonii	N	I	I
Ovis aries	N	I	I
Capra hircus	N	I	I
Odocoileus virginianus	N	I	I
Pteropus vampyrus	N	I	I
Callithrix jacchus	N	I	I
Cebus capucinus	N	I	I
Saimiri boliviensis	N	I	I
Carlito syrichta	N	I	I
Fukomys damarensis	N	I	I
Tupaia chinensis	N	I	I
Oryctolagus cuniculus	N	I	I
Cavia porcellus	N	I	I
Chinchilla lanigera	N	I	I
Peromyscus maniculatus	N	I	I
Cricetulus griseus	N	I	I
Microtus ochrogaster	N	I	I
Mus caroli	N	I	I
Mus musculus	N	I	I
Mus pahari	N	I	I
Mus spretus	N	I	I
Jaculus jaculus	N	I	I
Rattus norvegicus	N	I	I
Ictidomys tridecemlineatus	N	I	I
Marmota marmota	N	I	I
Nannospalax galili	N	I	I
Chlorocebus sabaeus	N	I	I
Colobus angolensis	N	I	I
Macaca fascicularis	N	I	I
Macaca nemestrina	N	I	I
Papio anubis	N	I	I
Mandrillus leucophaeus	N	I	I
Rhinopithecus bieti	N	I	I
Rhinopithecus roxellana	N	I	I
Piliocolobus tephrosceles	N	I	I
Macaca mulatta	N	I	I
Gorilla gorilla	N	I	I
Homo sapiens	N	I	I
Nomascus leucogenys	N	I	I
Pan paniscus	N	I	I
Pan troglodytes	N	I	I
Pongo abelii	N	I	I
Propithecus coquereli	N	I	I
Mesocricetus auratus	N	I	I
Dipodomys ordii	N	I	I
Sorex araneus	N	I	I
Acinonyx jubatus	N	I	L
Camelus ferus	N	I	I
Camelus dromedarius	N	I	I
Camelus bactrianus	N	I	I
Procavia capensis	N	I	I
Aotus nancymaae	N	I	I
Manis javanica	N	I	I
Ornithorhynchus anatinus	N	I	I
Cercocebus atys	N	I	I
Galeopterus variegatus	N	I	I

IRS1			
Nannospalax galili	N	T	T
Eptesicus fuscus	N	T	T
Myotis brandtii	N	T	T
Myotis myotis	N	T	T
Myotis lucifugus	N	T	T
Miniopterus natalensis	N	S	S
Sorex araneus	N	S	S
Ochotona princeps	N	S	S
Dasypus novemcinctus	N	S	S
Piliocolobus tephrosceles	N	S	S
Chrysochloris asiatica	N	S	S
Sarcophilus harrisii	N	S	S
Monodelphis domestica	N	S	S
Mus pahari	N	S	S
Callithrix jacchus	N	S	S
Aotus nancymaae	N	S	S
Lipotes vexillifer	N	S	S
Orcinus orca	N	S	S
Balaenoptera acutorostrata	N	S	S
Phascolarctos cinereus	N	S	S
Notamacropus eugenii	N	S	S
Ovis aries	N	S	S
Otolemur garnettii	N	S	S
Dipodomys ordii	N	S	S
Elephantulus edwardii	N	S	S
Mustela putorius	N	S	S
Mus caroli	N	S	S
Mus spretus	N	S	S
Rattus norvegicus	N	S	S
Manis javanica	N	S	S
Jaculus jaculus	N	S	S
Echinops telfairi	N	S	S
Equus caballus	N	S	S
Ceratotherium simum	N	S	S
Sus scrofa	N	S	S
Rhinolophus sinicus	N	S	S
Bos taurus	N	S	S
Bos mutus	N	S	S
Bos indicus	N	S	S
Bison bison bison	N	S	S
Bubalus bubalis	N	S	S
Pantholops hodgsonii	N	S	S
Capra hircus	N	S	S
Odocoileus virginianus	N	S	S
Equus asinus	N	S	S
Equus przewalskii	N	S	S
Camelus bactrianus	N	S	S
Camelus ferus	N	S	S
Camelus dromedarius	N	S	S
Hipposideros armiger	N	S	S
Choloepus hoffmanni	N	S	S
Tupaia chinensis	N	S	S
Cercocebus atys	N	S	S
Mandrillus leucophaeus	N	S	S
Rhinopithecus bieti	N	S	S
Homo sapiens	N	S	S
Pan paniscus	N	S	S
Nomascus leucogenys	N	S	S
Pan troglodytes	N	S	S
Gorilla gorilla	N	S	S
Macaca fascicularis	N	S	S
Macaca nemestrina	N	S	S
Macaca mulatta	N	S	S
Colobus angolensis	N	S	S
Ursus maritimus	N	S	S
Odobenus rosmarus	N	S	S
Neomonachus schauinslandi	N	S	S
Leptonychotes weddellii	N	S	S
Physeter catodon	N	S	S
Canis familiaris	N	S	S
Condylura cristata	N	S	S
Galeopterus variegatus	N	S	S
Ictidomys tridecemlineatus	N	S	S
Marmota marmota	N	S	S
Chinchilla lanigera	N	S	S
Fukomys damarensis	N	S	S
Heterocephalus glaber	N	S	S
Pteropus alecto	N	S	S
Rousettus aegyptiacus	N	S	S
Trichechus manatus	N	S	S
Peromyscus maniculatus	N	S	S
Microtus ochrogaster	N	S	S
Meriones unguiculatus	N	S	S
Mus musculus	N	S	S
Enhydra lutris	N	S	S
Cricetulus griseus	N	S	S
Mesocricetus auratus	N	S	S
Ailuropoda melanoleuca	N	S	S
Cavia porcellus	N	S	S
Octodon degus	N	S	S
Castor canadensis	N	S	S
Orycteropus afer	N	S	S
Loxodonta africana	N	S	S
Oryctolagus cuniculus	N	S	S
Felis catus	N	S	S
Panthera pardus	N	S	S
Acinonyx jubatus	N	S	S
Pteropus vampyrus	N	S	S
Delphinapterus leucas	N	A	A
Panthera tigris	N	W	W

TTI1			
Myotis brandtii	S	T	C
Myotis myotis	S	T	C
Eptesicus fuscus	S	T	C
Heterocephalus glaber	S	T	C
Myotis lucifugus	S	T	C
Bos indicus	S	A	C
Sus scrofa	S	A	C
Octodon degus	S	A	C
Phascolarctos cinereus	C	A	C
Saimiri boliviensis	S	A	C
Pongo abelii	S	A	C
Microcebus murinus	S	A	C
Choloepus hoffmanni	S	A	C
Loxodonta africana	S	A	C
Orycteropus afer	S	A	C
Tupaia chinensis	S	A	C
Tupaia belangeri	S	A	C
Bubalus bubalis	S	A	C
Chrysochloris asiatica	S	A	C
Dipodomys ordii	S	A	C
Cricetulus griseus	S	A	C
Mesocricetus auratus	S	A	C
Cavia porcellus	S	A	C
Fukomys damarensis	S	A	C
Monodelphis domestica	C	A	C
Sarcophilus harrisii	C	A	C
Notamacropus eugenii	C	A	C
Trichechus manatus	S	A	C
Jaculus jaculus	S	A	C
Nannospalax galili	S	A	C
Ailuropoda melanoleuca	S	A	C
Ursus maritimus	S	A	C
Neomonachus schauinslandi	S	A	C
Leptonychotes weddellii	S	A	C
Odobenus rosmarus	S	A	C
Felis catus	S	A	C
Panthera tigris	S	A	C
Enhydra lutris	S	A	C
Mustela putorius	S	A	C
Aotus nancymaae	S	A	C
Callithrix jacchus	S	A	C
Cebus capucinus	S	A	C
Mandrillus leucophaeus	S	A	C
Macaca fascicularis	S	A	C
Macaca mulatta	S	A	C
Macaca nemestrina	S	A	C
Papio anubis	S	A	C
Piliocolobus tephrosceles	S	A	C
Rhinopithecus bieti	S	A	C
Rhinopithecus roxellana	S	A	C
Colobus angolensis	S	A	C
Gorilla gorilla	S	A	C
Homo sapiens	S	A	C
Pan troglodytes	S	A	C
Nomascus leucogenys	S	A	C
Propithecus coquereli	S	A	C
Galeopterus variegatus	S	A	C
Carlito syrichta	S	A	C
Equus przewalskii	S	A	C
Equus asinus	S	A	C
Ceratotherium simum	S	A	C
Miniopterus natalensis	S	A	C
Eptesicus fuscus	S	A	C
Rousettus aegyptiacus	S	A	C
Pteropus vampyrus	S	A	C
Pteropus alecto	S	A	C
Camelus bactrianus	S	A	C
Camelus dromedarius	S	A	C
Camelus ferus	S	A	C
Vicugna pacos	S	A	C
Castor canadensis	S	A	C
Dasypus novemcinctus	S	A	C
Erinaceus europaeus	S	A	C
Bos taurus	S	A	C
Bison bison	S	A	C
Bos mutus	S	A	C
Pantholops hodgsonii	S	A	C
Ovis aries	S	A	C
Capra hircus	S	A	C
Odocoileus virginianus	S	A	C
Orcinus orca	S	A	C
Delphinapterus leucas	S	A	C
Physeter catodon	S	A	C
Balaenoptera acutorostrata	S	A	C
Manis javanica	S	A	C
Oryctolagus cuniculus	S	A	C
Ictidomys tridecemlineatus	S	A	C
Marmota marmota	S	A	C
Condylura cristata	S	A	C
Elephantulus edwardii	S	A	C
Meriones unguiculatus	S	A	C
Mus caroli	S	A	C
Mus musculus	S	A	C
Mus spretus	S	A	C
Rattus norvegicus	S	A	C
Peromyscus maniculatus	S	A	C
Microtus ochrogaster	S	A	C
Chinchilla lanigera	S	A	C
Procavia capensis	C	A	C
Sorex araneus	S	A	C

Figure S1. Other mammals share same amino acid sites with long-lived species in this study.

**Species share same amino acid sites with long-lived species are highlight in red. (the convergence sites between long-lived mammals and other mammals are marked in orange and the remaining are in blue).
